# Mating Signals Indicating Sexual Receptiveness Induce Unique Spatio-Temporal EEG Theta Patterns in an Anuran Species

**DOI:** 10.1371/journal.pone.0052364

**Published:** 2012-12-21

**Authors:** Guangzhan Fang, Ping Yang, Jianguo Cui, Dezhong Yao, Steven E. Brauth, Yezhong Tang

**Affiliations:** 1 Chengdu Institute of Biology, Chinese Academy of Sciences, Chengdu, Sichuan, China; 2 Key Laboratory for NeuroInformation of Ministry of Education, School of Life Science and Technology, University of Electronic Science and Technology of China, Chengdu, China; 3 Department of Psychology, University of Maryland, College Park, Maryland, United States of America; University of California, Riverside, United States of America

## Abstract

Female mate choice is of importance for individual fitness as well as a determining factor in genetic diversity and speciation. Nevertheless relatively little is known about how females process information acquired from males during mate selection. In the Emei music frog, *Babina daunchina*, males normally call from hidden burrows and females in the reproductive stage prefer male calls produced from inside burrows compared with ones from outside burrows. The present study evaluated changes in electroencephalogram (EEG) power output in four frequency bands induced by male courtship vocalizations on both sides of the telencephalon and mesencephalon in females. The results show that (1) both the values of left hemispheric theta relative power and global lateralization in the theta band are modulated by the sexual attractiveness of the acoustic stimulus in the reproductive stage, suggesting the theta oscillation is closely correlated with processing information associated with mate choice; (2) mean relative power in the beta band is significantly greater in the mesencephalon than the left telencephalon, regardless of reproductive status or the biological significance of signals, indicating it is associated with processing acoustic features and (3) relative power in the delta and alpha bands are not affected by reproductive status or acoustic stimuli. The results imply that EEG power in the theta and beta bands reflect different information processing mechanisms related to vocal recognition and auditory perception in anurans.

## Introduction

Mating choices affect the fitness of individuals and contribute to evolutionary diversity and are thus among the most important decisions made by organisms [1]. During mate choice, females are believed to discriminate among males on the basis of genetic quality and the resources offered to the female or her offspring. Ultimately females typically reject all but the last male considered [2]. Previous studies provide considerable information about the strategies used by females to choose mates [3]–[10], however, we still know little about the neural underpinnings of female choice, especially the process of information acquisition and processing.

Because the electroencephalogram (EEG) allows broad canvassing of brain areas composed of multiple neuronal populations which might be involved in different functions, changes in power output within EEG bands were used to investigate the neural bases of information processing related to mate choice in an anuran species, the Emei music frog, *Babina daunchina,* in the present study. This frog was selected because its vocal communication is well suited to studying female choice [11], [12]. *B. daunchina* males produce advertisement calls during breeding seasons both from within nest burrows the male has made and from outside the burrows. The resonant properties of the burrow alter some of the call acoustics and phonotaxis experiments show that females in the reproductive stage prefer male calls produced from inside burrows [12]. In the present study calls recorded from males within burrows were regarded as highly sexually attractive (HSA) calls while those produced outside the burrow were regarded as low sexually attractive (LSA) calls.

The EEG was used to assess brain activity in this species because many studies have shown that power within EEG frequency bands are typically correlated with specific brain states and predominate during different behavioral states [13]–[16] such as sensory registration, perception, movement and cognitive processes related to attention, learning, memory and decision making [14], [17]–[20]. Moreover, the high time resolution of the EEG enables correlation of physiological responses with the occurrence of discrete sensory stimuli including biologically relevant stimuli such as courtship vocalizations.

EEG recordings were made from the mesencephalon (midbrain) and telencephalon (cerebrum) in the present study. Frogs have a relatively well developed auditory midbrain (the torus semicircularis) which is thought to be homologous to the auditory midbrain of amniotes and has been shown to be critically involved in acoustic information processing [21]–[24]. In addition while no direct counterparts to the specific sensory telencephalic areas of amniotes have been identified in the anuran forebrain, widespread connections exist linking forebrain neurons to motor, endocrine, and a variety of limbic structures in frogs [25]. It is thus of considerable scientific importance to determine how the anuran forebrain processes complex sensory signals, such as courtship vocalizations. Burmeister and colleagues showed that conspecific call stimuli evoked immediate early gene expression in both torus semicircularis and dorsal medial and ventral pallium in frogs. They hypothesized that neural activity in the torus and pallium could be involved in regulating acoustically mediated sexual behavior [24], [26]. The present study was also designed to investigate this hypothesis further using electrophysiology.

In general, mate choice studies have shown that strict preferences tend to guide female decision making such that all properties of a stimulus can be reduced to a single preference value independent of other stimuli [9]. Thus, we predicted that the EEG of female frogs would reflect the degree to which stimuli are biologically relevant. Specifically we predicted that females exhibit corresponding EEG patterns in response to various acoustic signals that indicate differing levels of sexual attractiveness in the reproductive stage. To test our predictions, we recorded multi-channel electrocorticogram (ECoG) signals from female *B. daunchina* in both the reproductive and non-reproductive stages in response to silence, a white noise (WN) stimulus, HSA and LSA calls recorded from the same male. Signal processing techniques were used to evaluate EEG responses to these acoustic stimuli across multiple frequency bands in the telencephalon and mesencephalon.

## Materials and Methods

### Animals

Eight female adult frogs (*Babina daunchina*) captured from the Emei mountain area of Sichuan, China in July 2011 were used in these experiments. Subjects were housed in an opaque plastic tank (45×35 cm and 30 cm deep) containing mud and water. The tank was placed in a room under controlled temperature conditions (23±1°C) and maintained on a 12∶12 light-dark cycle (lights on at 08∶00 h). The animals were fed fresh live crickets every three days. Mean masses were 12.0±2.2 g (mean ± SD), and frogs were 4.9±0.2 cm in length at the time of surgery. Permits were obtained from Department of Forestry Management, Mount Emei and Leshan Giant Buddha Management Committee. All experiments were approved by the Chengdu Institute of Biology Animal Care and Use Committee (Permit Number: 2011–015) and carried out according to the international standards of animal care and use. All surgery was performed under sodium pentobarbital anesthesia, and all efforts were made to minimize suffering.

### Surgery

**Figure 1:**
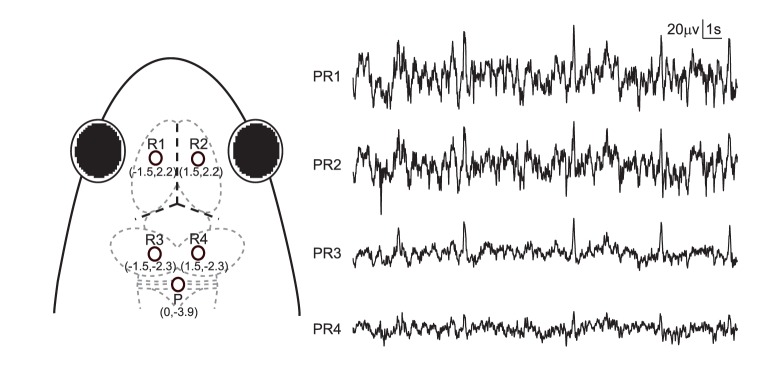
Electrode placements and 20 s of typical EEG tracings for each channel. The intersection of the three dashed lines in bold in the frog head denotes the intersection of suture lines corresponding to lambda.

All experiments were conducted after September 2011 when the reproductive season ended for the frogs [11]. Surgical procedures are described in detail in our previous study [27]. In short, sterile surgery was performed under deep anesthesia induced by intraperitoneal pentobarbital sodium (3 mg/100 g). Then, four cortical EEG electrodes, composed of miniature stainless steel screws (ϕ 0.8 mm), were implanted on the frog skull: the left and right sides of the telencephalon and mesencephalon (R1, R2, R3 and R4), and referenced to the electrode above the cerebellum (P, the corresponding electrode pairs were abbreviated as PR1, PR2, PR3 and PR4, respectively) (Fig. 1). Twenty seconds of typical EEG waves were presented along with the corresponding electrode pairs (Fig. 1). R1 and R2 were implanted bilaterally 2.2 mm anterior to the lambda (i.e. the point where skull sutures intersect) and 1.5 mm lateral to the midline, respectively, while R3 and R4 were implanted bilaterally 2.3 mm posterior to the lambda and 1.5 mm lateral to the midline. P was implanted 3.9 mm posterior to the lambda at the midline. One end of a formvar-insulated (except at the two ends) nichrome wire (ϕ 0.1 mm) served as a ground electrode and was fixed subcutaneously about 5 mm posterior to P.

All electrode leads were formvar-insulated nichrome wires with one end interwined tightly around the miniature stainless steel screws except for the ground electrode which was fixed subcutaneously and directly, and with the other end tin soldered to the female-pins of an electrical connector (GongZhan, PHD2004S; DongGuang, China). Electrodes were fixed to the skull with dental acrylic. The connector was covered with self-sealing membrane (Parafilm® M; Chicago, USA) for water-proofing and located about 1 cm above the head of the animal. For best suspension, six formvar-insulated nichrome wires were wound together around a pencil to form a loose spring [28]. One end of each wire was tin soldered to the male-pins of the electrical connector; the other ends were connected to the cable of the signal acquisition system (Chengyi, RM6280C; Sichuan, China). The junction points were kept in a box covered with silver paper and earthed.

Each frog was housed singly for 2 days for recovery before the following experiments were performed. After the end of the experiments, all frogs were euthanized by overdose of intraperitoneal pentobarbital sodium and electrode localizations were confirmed by injecting hematoxylin dye through the skull holes in which the electrodes were installed previously.

### Stimulus Presentation

Four stimuli were used in the current study: HSA call, LSA call, WN and silence. To eliminate individual effects, both HSA and LSA calls containing five notes recorded from the same individual were selected randomly from the dataset of our laboratory [12]. WN was constructed as a consecutive “call” and its duration was the average of durations of the HSA and LSA calls (about 1.2 s), containing a 15 ms period with sinusoidal rise-fall characteristics. In addition, silence is also a usual form of acoustic ambience which animals encounter in their natural environment. When given the choice between ethologically relevant sounds (including silence) or other sounds (including music or WN), animals will often prefer the former [29], [30]. In other words, silence is also biologically relevant for animals; therefore, it was used as a stimulus in the present study.

### Data Acquisition

The experiments were performed in a soundproof and electromagnetic shielded chamber in which the background noise was 23.0±1.7 dB (mean ± SD) with an opaque plastic tank (80×60 cm and 55 cm deep) containing mud and water at the center. Lights and temperature in the chamber were maintained as in the home-cages. A video camera with infrared light source and motion detector was appended centrally above the tank about 1 m for monitoring the behavior of the subject from outside of the chamber. In order to eliminate the effects of digestion on the results, the subject was not fed during the experimental period.

The procedure for data collection consisted of electrophysiological and behavioral recordings in four sequential days: on the first day the subject was placed in the experiment tank and connected to the signal acquisition system for habituation; neurophysiological and behavioral data were collected for the subject in the non-reproductive stage on the second day; the animal was then administered gonadotropin-releasing hormone (GnRH-A6; Sichuan, China; i.p. 1.25 µg per frog at 21∶30 h) on the third day, and neurophysiological and behavioral data were acquired for the frog in the reproductive stage on the fourth day. On the second and fourth days, the reproductive status of the subject was determined behaviorally by phonotaxis experiments [12].

For playbacks, two portable field speakers (SME-AFS, Saul Mineroff Electronics, Elmont, NY, USA) were placed equidistantly from the opposite ends of the tank. Two speakers were set 1.7 m apart, outside of the tank with a rectangular hole (20×15 cm) at the central wall area of each end. The four stimuli (silence, HSA call, LSA call and WN) were presented to females in a randomized sequence, in which each stimulation continued for 10 minutes with 4 s inter-stimulus intervals between each call presentation, approximately equal to the mean of inter-call intervals of *B. daunchina* [12]. Each 10 minute stimulation period except for the silence stimulus followed by an hour silent period, and all stimuli except for silence were equalized for intensity (65 dB SPL, re 20 mPa; Aihua, AWA6291; Hangzhou, China; measured at the centre of the tank). A trigger pulse was sent to the signal acquisition system at each stimulus onset through the parallel port. Before the experiments, we used a 1000-Hz tone to calibrate the peak output intensity of each speaker to 70 dB SPL (measured at the centre of the tank).

*B. daunchina* remains active throughout the day except for the periods around noon and midnight [27], with the highest level of activity occurring at dawn and dusk. For this reason the experiments began at 20∶30 on the second and fourth days. In this way, the subjects remained awake during the experiments, yet produced few movements which could cause EEG artifacts. The signal acquisition system was set to record continuously from the start of the experiments to the end. Bandpass filters, set to 0.16–100 Hz, were used for EEG signals with the notch filter of the amplifiers set to eliminate possible interference at 50 Hz. A sampling frequency of 1000 Hz was used.

### Data Processing

Prior to power spectrum analysis, for each frog and each channel, representative and artifact-free segments (100 s) were selected randomly from electrophysiological data collected on the second and fourth days, respectively. The same time windows were adopted in selecting data for all the four channels. After band-pass filtering (0.5–45 Hz) and downsampling at 512 Hz, these segments were divided into 5 s epochs for each channel and each day, each of which was detrended (i.e. the linear trend was removed) using an algorithm which computes the least-squares fit of the data. Standard deviations were calculated for each epoch, each channel and each day.

Because females usually make a choice in approximately 4 minutes (220±134 s) in phonotaxis experiments, only the first 5 minutes of EEG data were analyzed for each stimulus. For this EEG segment for each stimulus, the data preprocessing was the same as that for the representative waves. For each channel and each day, those epochs whose standard deviations were 3 times as large as the maximum standard deviation of the representative waves were discarded as artifacts. The designation of artifact in any one channel resulted in the removal of data in all channels to ensure that data preserved in all channels were derived from the identical time periods. For artifact-free epochs, the power spectra of delta (0.5–5.5 Hz), theta (5.5–8.5 Hz), alpha (8.5–17 Hz) and beta (17–45 Hz) were computed by Welch’s method with the Hamming window. The resolution of the power spectrum was set at 0.5 Hz and the boundaries of the EEG frequency bands were determined on the basis of the results of factor analysis of EEG in frogs [27].

Many studies calculate both absolute and relative EEG power, and have found that the use of absolute power can yield different results than those obtained using relative power [31]–[33]. Because relative power values exhibit lower group variability than absolute power values and are not affected by the influences of EEG amplitude depending on the electrical volume conductor properties of the head [31], the use of relative power is more suitable for comparison between different channels. For these reasons, only relative power (band power/total power across bands) computed over the entire data epoch (5 minutes) was used to determine the percent of brain activity within a given band for each stimulus, each channel, each reproductive status and each frog.

To explore the temporal properties of the EEG, EEGLAB software was used to run time-frequency analyses in which spectral power was measured at different points in time during an epoch, using wavelet analysis [34]. EEG recordings were initially band-pass filtered (Low Pass Filter cutoff frequency: 45 Hz, High Pass Filter cutoff frequency: 0.25 Hz) and were then separated into non-overlapping epochs time-locked to each of the four acoustical stimuli onset (including silence). The period from −200 to 0 ms before stimulus presentation served as the baseline [35]. Epochs containing high amplitude, high-frequency muscle noise and other irregular artifacts, as identified by visual inspection, were removed. The superimposed average waveform across-trial was calculated for each stimulus, each channel and each subject. Then, the grand averages of these waveforms across subjects were used to detect transient event-related spectral perturbation (ERSP). The analysis and extraction of the artifacts was performed off-line on a PC by means of the EEGLAB software coded in MATLAB. The default settings were used.

### Statistical Analyses

The normality of the distribution of the values was tested using the Schapiro-Wilk W test. If the values were not normally distributed, they were logit transformed before statistical analyses [33], [36]. To evaluate differences in EEG power, 3-way within-subject ANOVA (i.e. 3-way repeated measures ANOVA) with the factors of “reproductive status”, “acoustic stimulus” and “electrode placement” was employed for each EEG band. Both main effects and interactions were examined. Simple effects analysis would be applied when the interaction was significant. For significant ANOVAs, data were further analyzed for multiple comparisons using the least-significant difference (LSD) test. Greenhouse-Geisser epsilon (ε) values were employed when the Greenhouse-Geisser correction was necessary. Estimations of effect size for ANOVAs were determined with partial 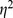 (partial 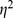 = 0.20 is a small effect size, 0.50 is a medium effect size and 0.80 is a large effect size) [37]. SPSS software (release 13.0) was utilized for the statistical analysis. A significance level of *p*<0.05 was used in all comparisons; *p* values >0.05 and <0.1 were considered as marginally significant [38].

## Results

### Significant Differences Occurred Only for the Theta and Beta Bands

**Figure 2:**
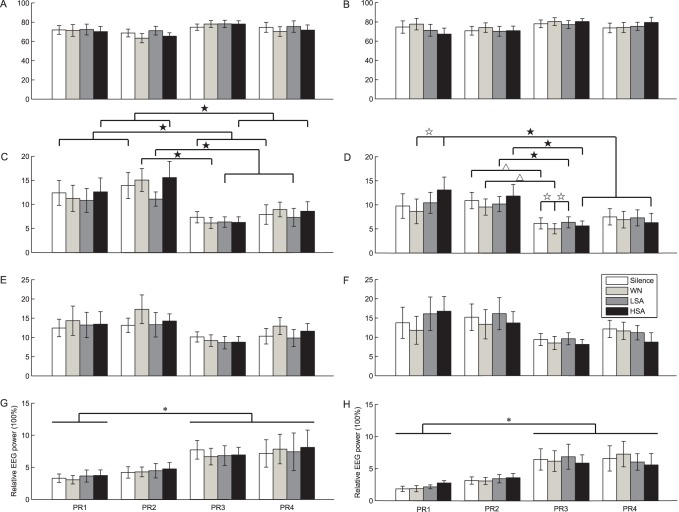
Means and standard errors of relative EEG power spectra. The four rows are for delta (A, B), theta (C, D), alpha (E, F) and beta (G, H) EEG bands, respectively; while the two columns are for the non-reproductive stage (left) and the reproductive stage (right), respectively. Filled stars or open triangles denote that there were significant or marginally significant differences between the corresponding electrode pairs during playback of a given acoustic stimulus (*p*<0.05 for filled stars and 0.05<*p*<0.1 for open triangles), open stars denote that there were significant differences between the corresponding acoustic stimuli for a given channel (*p*<0.05), while asterisks denote that relative EEG power for the mesencephalon (PR3 and PR4) were significantly higher than that for the left telencephalon (PR1) (*p*<0.05). PR1, PR2, PR3 and PR4, the four electrode pairs; WN, white noise; HSA, high sexual attractive call; LSA, low sexual attractive call.

**Figure 3:**
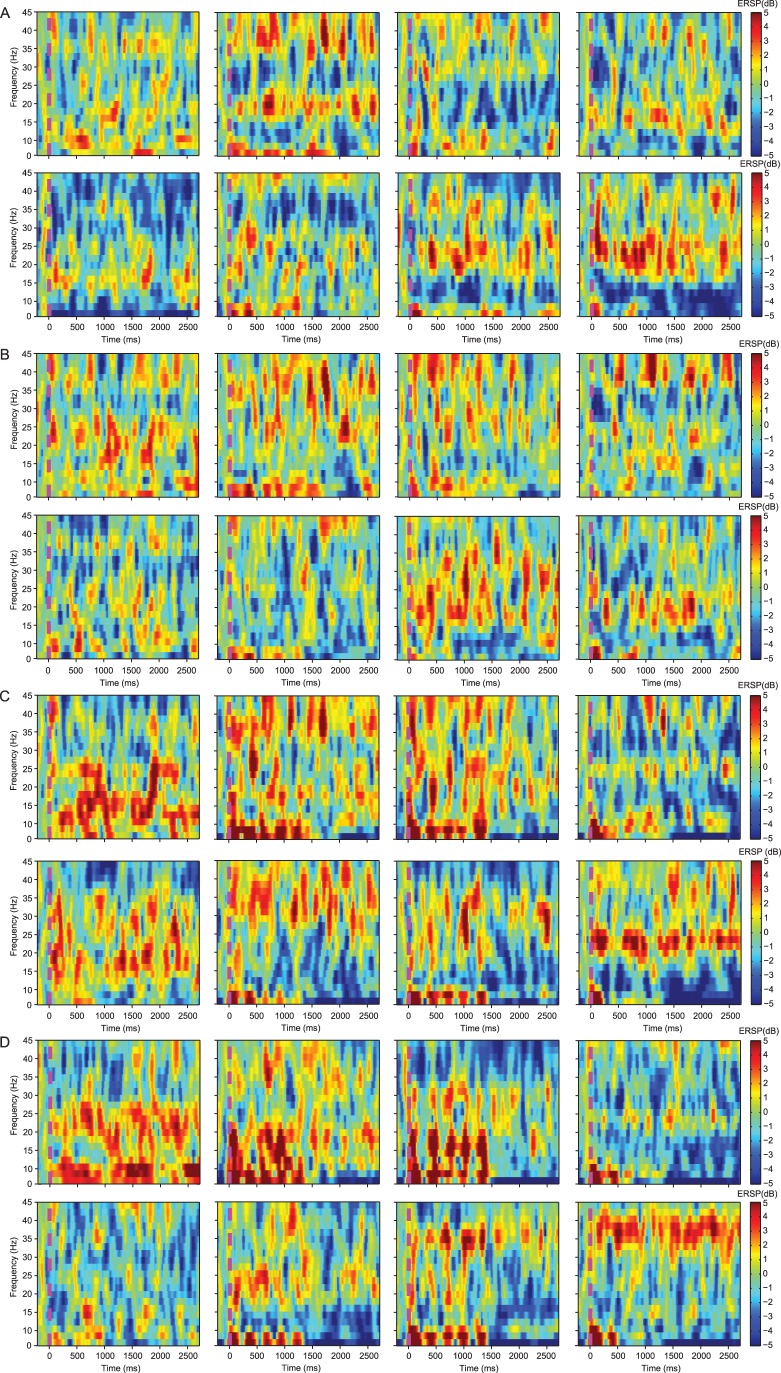
Time-frequency maps of grand mean EEG waveforms across subjects. The four subplots are for the left (A) and right (B) telencephalon, the left (C) and right (D) mesencephalon, respectively. For each subplot, the two rows are for the non-reproductive stage (upper) and the reproductive stage (lower), respectively; while the four columns are for the four stimuli, i.e. silence, white noise, low sexual attractive call and high sexual attractive call. Only the data from −200 to 2700 ms for each stimulus onset are shown in the figure. The pink vertical dotted line denotes the onset of a given stimulus. ERSP, event-related spectral perturbation.

For the delta and alpha bands, the results of ANOVA showed that all main effects and interactions did not reach statistical significance (*p*>0.05) (Fig. 2A, 2B, 2E and 2F), suggesting that relative power in these two bands was not affected by the variables considered in this study, although time-frequency analysis showed that the activities in the alpha band was lower than for the other EEG bands under LSA and HSA calls in the reproductive stage (Fig. 3). For the beta band, the main effect for the factor “electrode placement” was significant (F (3, 21) = 5.326; *p*<0.05, partial 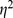 = 0.432) (Fig. 2G and 2H), and multiple comparisons showed relative beta power of the mesencephalon (PR3 and PR4) was significantly higher than that of the left telencephalon (PR1, *p*<0.05). The beta band response to acoustic stimuli did not change with reproductive status or the biological significance of signals. For the theta band, the results of ANOVA revealed that both the main effects were significant for the factor “reproductive status” (F (1, 7) = 6.298; *p*<0.05, partial 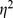 = 0.474) and the factor “electrode placement” (F (3, 21) = 5.849; *p*<0.05, partial 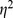 = 0.455), respectively; the interaction between “electrode placement” and “acoustic stimulus” was significant (F (9,63) = 2.174; *p*<0.05, partial 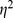 = 0.237) (Fig. 2C and Fig. 2D). Because the interaction was significant, simple effect analysis was further applied.

### Spatiotemporal Theta Patterns were Similar for Different Acoustic Stimuli in the Non-reproductive Stage

In the non-reproductive stage, all four acoustic stimuli yielded undifferentiated theta power for each recording electrode site (*p*>0.05, Fig. 2C and Table 1) implying that non-reproductive females process the four acoustic signals similarly. Simple effect analysis showed that global lateralization for the theta band was similar during playbacks of different acoustical stimuli in this stage. The relative theta power of the telencephalon (PR1 and PR2) was significantly higher than those of the mesencephalon (PR3 and PR4) during silence and playback of HSA calls (*p*<0.05). Relative theta power of the right telencephalon (PR2) was significantly higher than that of the right mesencephalon (PR3) during playback of WN (*p*<0.05). Relative theta power of the right telencephalon (PR2) was significantly higher than those of the mesencephalon (PR3 and PR4) during playback of LSA calls (*p*<0.05, Fig. 2C and Table 1).

**Table 1:** Results of simple effect analysis for the factors “channel” and “stimulus”.

	Non-reproductive stage				Reproductive stage			
Factor	F(3,21)	ε	*p*	LSD	F(3,21)	ε	*p*	LSD
*channel*								
PR1	2.263	0.623	0.145	NA	3.951	NA	0.022*	HSA>WN
PR2	1.689	NA	0.2	NA	2.746	0.479	0.121	NA
PR3	1.754	NA	0.187	NA	3.93	NA	0.023*	SIL,LSA>WN
PR4	1.252	NA	0.316	NA	0.796	NA	0.51	NA
*stimulus*								
SIL	6.072	0.531	0.021*	PR1,2>PR3,4	3.436	0.603	0.068#	PR2>PR3
WN	4.809	NA	0.011*	PR2>PR3	2.555	NA	0.083#	PR2>PR3
LSA	4.951	NA	0.009*	PR2>PR3,4	3.176	NA	0.045*	PR2>PR3
HSA	5.263	0.476	0.035*	PR1,2>PR3,4	7.004	NA	0.002*	PR2>PR3
								PR1>PR3,4

*Note:* The symbols ‘>’ denote that the relative theta power given by the channels or stimuli on the left side of ‘>’ are significantly larger than those given by the channels or stimuli on the right side, and no significant difference exists among the channels or stimuli at the same side of ‘>’ for each case.

*p<0.05,

# *p<*0.1 (marginally significant). Abbreviations: *F* is the *F*-value from ANOVA; ε, the values of epsilon of Greenhouse-Geisser correction; LSD, the least-significant difference test; PR1, PR2, PR3 and PR4, the four channels; SIL, WN, LSA and HSA, the four stimuli, i.e. silence, white noise, low sexual attractive call and high sexual attractive call.

### Spatiotemporal Theta Patterns were Modulated by the Sexual Attractiveness of the Acoustic Stimuli in the Reproductive Stage

There were significant differences in theta power among the four acoustic stimuli in the left but not the right hemisphere in the reproductive stage (Table 1) and activities in the beta and low-frequency (including theta band) oscillations were obviously higher than for the alpha band under LSA and HAS calls for each channel, especially in the left hemisphere (Fig. 3). These results are consistent with the idea that spatio-temporal EEG patterns are modulated by the sexual attractiveness of acoustic stimulus in the reproductive stage. Relative theta power during playback of HSA calls was significantly higher than during playback of WN for the left telencephalon (PR1, *p*<0.05); while relative theta power during silence and playback of LSA calls were significantly higher than during playback of WN for the left mesencephalon (PR3, *p*<0.05) (Fig. 2D and Table 1). Global lateralization for the theta band was modulated by the sexual attractiveness of the acoustic stimulus in the reproductive stage. Theta power in the right telencephalon (PR2) was significantly higher than in the left mesencephalon (PR3) under HSA call playback (*p*<0.05), LSA call playback (*p*<0.05), WN (marginally significant, *p* = 0.083<0.1) and conditions of silence (marginally significant, *p* = 0.068<0.1). Furthermore theta power in the left telencephalon (PR1) was significantly higher than for the mesencephalon (PR3 and PR4) under HSA call playback.

As described above, relative power in the theta and beta bands was significantly affected by one or more factors considered in this study. Furthermore, the results from the time-frequency analysis show that the beta and low-frequency EEG oscillations including the theta band reflect neural processing of mating signals, especially in the left hemisphere (Fig. 3). Previous studies have shown that the theta oscillation can reflect cognitive processing including decision making [39] while the beta oscillation has been related to sensorimotor functions [40]. As discussed below, the data presented in the present study suggests these ideas can be extended to the acquisition and processing of information related to courtship vocalizations in the music frog.

## Discussion

Relative power in the theta and beta bands was significantly affected by one or more factors including reproductive status, electrode placement and the type of acoustic stimulus presented, whereas power in the delta and alpha bands was not influenced by these variables. For the theta band, both global lateralization and the values of left hemispheric relative power were modulated by the sexual attractiveness of the acoustic stimulus in the reproductive stage. In contrast, relative power in the beta band to acoustic stimuli did not change with reproductive status or the biological significance of signals and was higher in the mesencephalon than in the left telencephalon. These results suggested that EEG power in the theta and beta bands reflect different processes related to acoustic signal processing with the theta band reflecting decision making processes closely related to mate choice.

### Theta Oscillation is Involved in Vocal Recognition

Previous studies on frogs have shown that the theta band is a spontaneous rhythmic pattern generated in the medial pallium [41], [42] which is considered to be homologous to the mammalian hippocampus [43], [44], and the predominant normal pattern of activity which can be recorded from the telencephalon [45], [46]. Moreover, the theta oscillation plays a basic role in cognitive processing and cortico- hippocampal interaction [19], and serves integrative functions in coordinating brain activity [47] as a universal operator involved in many cognitive functions [39] including information coding, attention, working memory maintenance and emotional regulation. Such functions would seem indispensable for survival and reproductive success in environments where the distribution and availability of resources frequently change.

In the non-reproductive stage, there were no significant differences of theta power among the four acoustic stimulus conditions, and the brain tended to process information using whole EEG frequency components (Fig. 3), suggesting that non-reproductive females process the four acoustic signals similarly. This idea is consistent with a previous behavioral study showing that the responses of anuran females to mating stimuli is modulated by estrogen levels [48]. In contrast, in the reproductive stage the four acoustic stimuli engendered significant differences in theta power in the left but not the right hemisphere. Time-frequency analysis also showed that the brain might combine theta and beta to process mating signals, especially in the left hemisphere (Fig. 3). For *B. daunchina*, conspecific calls and silence can be regarded as biologically relevant sounds while WN is not biologically relevant. Thus left hemisphere differences in the theta response might reflect information processing associated with the sexual attractiveness of signals. The same pattern of dominance in the left hemisphere for processing species-specific communication sounds has been reported in mice, rats, passerine birds, primates and humans [49], [50]. The present study provides direct evidence that a seemingly analogous processing mechanism for biologically relevant sensory cues associated with mate choice is also lateralized in anurans.

It has been demonstrated that the left hemisphere is more frequently associated with approach behavior and positive affect while the right hemisphere is more frequently associated with avoidance behavior, behavioral inhibition and negative affect [51]–[54]. Our study reveals statistically significant differences in theta power between WN and HSA call in the left telencephalon and between WN and LSA call in the left mesencephalon. These differences may reflect the fact that HSA or LSA calls compared with WN are likely to induce approach responses for *B. daunchina* subjects [12]. Thus, theta power recorded from the left hemisphere (PR1 and PR3) during WN playback was generally significantly lower than for all other acoustic stimulus conditions (although some differences did not reach statistical significance), implying a special role for the left hemisphere including the left pallium in *B. daunchina* for processing stimuli associated with mate selection.

The significant differences in theta power among the recording channels were largely different for each of the four acoustic stimuli in the reproductive stage. In contrast, significant differences in theta power output among electrode channels were similar for all four stimuli in the non-reproductive stage. These results show that the spatiotemporal patterns of theta oscillation were modulated by both reproductive status and the type of acoustic stimulus presented. Of particular interest, the channel differences for the theta band under conditions of silence in the non-reproductive stage decreased to only the marginally significant level for frogs in the reproductive stage, while whole brain activities under silence in the non-reproductive stage also decreased when subjects were in the reproductive stage (Fig. 3). These results imply that in the reproductive stage the subjects attend selectively to stimuli associated with breeding while in the non-reproductive stage subjects attend to a broader range of stimuli which may be useful for survival. The concept of self-organized criticality (SOC) [55], [56] may apply for explaining the reduced (and only marginally significant) level under conditions of silence in the reproductive stage, in other words, the brain approaches and remains in a critical state at this time. Such a shift to a critical state seems to manifest itself as differences in the spatiotemporal pattern of lateralization of the theta response to HSA or LSA calls vs WN in the reproductive stage.

The theta oscillation coordinates brain activity in different subregions and layers of the hippocampus to support behavioral task performance [57], and reflects the encoding of new information in hippocampo-cortical feedback loops [14]. Furthermore, the theta oscillation has been proposed to code phase in working memory [47] and to form the recurrent interactions between neurons in different brain regions that underlies working memory [58]. Female frogs choose mates based on adequate assessments in which females maintain attention to vocal signals via working memory [59], while memory information is stored within a distributed theta network and matched with an incoming sensory trace in posterior brain areas [60]. The integration between top-down processes guided by a complex working memory system and the bottom-up processing of perceptual information may be reflected by a dynamic interaction between theta and high frequency oscillations [60]. This integration might be closely related with decision making which involves at least two general stages of neural processing: representation of evidence from early sensory areas and accumulation of evidence to a decision threshold from decision-related regions [61]. Taken together, these results indicate that the theta modulation including presenting in the telencephalon might play a critical role in vocal information processing associated with mate choice in frogs.

### Beta Oscillation is Related to Acoustic Signal Processing

The EEG beta band contains the highest frequency components recorded on the subjects and has long been considered to be related to sensorimotor functions [40] or cognitive state [62]. In the present study, the beta oscillation differed from theta in two ways. First beta power output in the mesencephalon was significantly higher than that of the left telencephalon, a pattern which did not differ between the non-reproductive and reproductive stages. These results suggest that this high frequency EEG component is not uniquely associated with information processing related to reproduction, and is consistent with the fact that beta is associated with many perceptual, motor, emotional and cognitive processes in humans [63], [64]. The time-frequency spectrogram showed that beta activities were higher in response to acoustic stimuli than in silence (before acoustic signal onset) in either the reproductive stage or the non-reproductive stage, suggesting the beta oscillation is involved non-selectively in the perception and processing of acoustic inputs. This is consistent with the fact that the auditory midbrain, the torus semicircularis, forms a large portion of the anuran midbrain, and has been shown to receive ascending auditory input from the brainstem as well as input from the forebrain and also as an audiomotor interface [25]. In view of these neuroanatomical considerations, we propose that the frog midbrain EEG beta oscillation reflects ongoing general sensorimotor functions.

Because there is no gamma band in the frog EEG [27], the beta band may play a special role in the maintenance of attention to environmental stimuli [65]. The dynamic integration of top-down and bottom-up processing of perceptual information in the mammalian cortex [60] has been proposed to reflect either modulation of fast neural activity in local circuits by large-scale networks operating at lower frequencies (top-down integration), or a bottom-up mechanism (higher frequencies) for propagation of local activation to other cortical regions [65]. In view of the results of the present study it appears likely that the beta oscillation reflects the processing of acoustic features in frogs, regardless of the frog’s reproductive status or the biological significance of the acoustic signals. Furthermore, higher frequency oscillations are confined to a small neuronal space [65], [66], thus higher beta power in the mesencephalon might reflect the demands of the rapid processing of acoustic signals for perception that not only encode mate and/or rival clues but also convey information of broad significance such as information concerning predators.

## References

[1] Phelps SM, Rand AS, Ryan MJ (2006) A cognitive framework for mate choice and species recognition. American Naturalist 167: 28–42. 10.1086/498538 16475097

[2] Moore AJ, Moore PJ (1988) Female strategy during mate choice: threshold assessment. Evolution 42: 387–391. 28567843 10.1111/j.1558-5646.1988.tb04141.x

[3] Janetos AC (1980) Strategies of female mate choice: a theoretical analysis. Behavioral Ecology and Sociobiology 7: 107–112.

[4] Parker G (1983) Mate quality and mating decisions. In: Bateson P, editor. Mate Choice. Cambridge (UK): Cambridge University Press. 141–164.

[5] Wittenberger JF (1983) Tactics of mate choice. In: Bateman P, editor. Mate Choice. Cambridge: Cambridge University Press. 435–447.

[6] Gibson RM, Langen TA (1996) How do animals choose their mates? Trends in Ecology & Evolution 11: 468–470. 21237923 10.1016/0169-5347(96)10050-1

[7] Alexander RD, Marshall DC, Cooley JR (1997) Evolutionary perspectives on insect mating. In: Choe JC, Crespi BJ, editors. The Evolution of Mating Systems in Insects and Arachnids. Cambridge (UK): Cambridge University Press. 4–31.

[8] Bateson M, Healy SD (2005) Comparative evaluation and its implications for mate choice. Trends in Ecology & Evolution 20: 659–664. 16701454 10.1016/j.tree.2005.08.013

[9] Kirkpatrick M, Rand AS, Ryan MJ (2006) Mate choice rules in animals. Animal Behaviour 71: 1215–1225.

[10] Leonard AS, Hedrick AV (2009) Male and female crickets use different decision rules in response to mating signals. Behavioral Ecology 20: 1175–1184.

[11] Cui JG, Wang YS, Brauth SE, Tang YZ (2010) A novel female call incites male-female interaction and male-male competition in the Emei music frog, *Babina daunchina* . Animal Behaviour 80: 181–187.

[12] Cui JG, Tang YZ, Narins PM (2011) Real estate ads in Emei music frog vocalizations: female preference for calls emanating from burrows. Biology Letters. doi: 10.1098/rsbl.2011.1091. 10.1098/rsbl.2011.1091 PMC3367746 22158736

[13] Engel AK, Fries P, Singer W (2001) Dynamic predictions: Oscillations and synchrony in top-down processing. Nature Reviews Neuroscience 2: 704–716. 11584308 10.1038/35094565

[14] Klimesch W (1999) EEG alpha and theta oscillations reflect cognitive and memory performance: a review and analysis. Brain Research Reviews 29: 169–195. 10209231 10.1016/s0165-0173(98)00056-3

[15] Csicsvari J, Jamieson B, Wise KD, Buzsaki G (2003) Mechanisms of gamma oscillations in the hippocampus of the behaving rat. Neuron 37: 311–322. 12546825 10.1016/s0896-6273(02)01169-8

[16] Kopell N, Ermentrout G, Whittington M, Traub R (2000) Gamma rhythms and beta rhythms have different synchronization properties. Proceedings of the National Academy of Sciences of the United States of America 97: 1867–1872. 10677548 10.1073/pnas.97.4.1867 PMC26528

[17] Thut G, Miniussi C (2009) New insights into rhythmic brain activity from TMS-EEG studies. Trends in Cognitive Sciences 13: 182–189. 19286414 10.1016/j.tics.2009.01.004

[18] Basar E, Basar-Eroglu C, Karakas S, Schurmann M (2000) Brain oscillations in perception and memory. International Journal of Psychophysiology 35: 95–124. 10677641 10.1016/s0167-8760(99)00047-1

[19] Basar E, Basar-Eroglu C, Karakas S, Schurmann M (2001) Gamma, alpha, delta, and theta oscillations govern cognitive processes. International Journal of Psychophysiology 39: 241–248. 11163901 10.1016/s0167-8760(00)00145-8

[20] Jacobs J, Hwang G, Curran T, Kahana MJ (2006) EEG oscillations and recognition memory: Theta correlates of memory retrieval and decision making. Neuroimage 32: 978–987. 16843012 10.1016/j.neuroimage.2006.02.018

[21] Feng AS, Shofner WP (1981) Peripheral basis of sound localization in anurans. Acoustic properties of the frog’s ear. Hearing Research 5: 201–216. 6975773 10.1016/0378-5955(81)90046-0

[22] Feng AS, Hall JC, Gooler DM (1990) Neural basis of sound pattern recognition in anurans. Progress in Neurobiology 34: 313–329. 2185497 10.1016/0301-0082(90)90008-5

[23] Edwards CJ, Alder TB, Rose GJ (2002) Auditory midbrain neurons that count. Nature Neuroscience 5: 934–936. 12219094 10.1038/nn916

[24] Burmeister SS, Mangiamele LA, Lebonville CL (2008) Acoustic modulation of immediate early gene expression in the auditory midbrain of female túngara frogs. Brain Research 1190: 105–114. 18061149 10.1016/j.brainres.2007.11.008

[25] Wilczynski W, Endepols H (2006) Central auditory pathways in anuran amphibians: the anatomical basis of hearing and sound communication. In: Narins PM, Feng AS, Fay RR, Popper AN, editors. Hearing and Sound Communication in Amphibians. Berlin, Germany: Springer. 221–249.

[26] Mangiamele LA, Burmeister SS (2008) Acoustically evoked immediate early gene expression in the pallium of female túngara frogs. Brain, Behavior and Evolution 72: 239–250. 10.1159/000171481 18997464

[27] Fang GZ, Chen Q, Cui JG, Tang YZ (2012) Electroencephalogram bands modulated by vigilance states in an anuran species: a factor analytic approach. Journal of Comparative Physiology A, Sensory, Neural, and Behavioral Physiology 198: 119–127. 10.1007/s00359-011-0693-y 22045113

[28] Laming P (1982) Electroencephalographic correlates of behavior in the anurans *Bufo regularis* and *Rana temporaria* . Behavioral and Neural Biology 34: 296–306. 6980644 10.1016/s0163-1047(82)91678-8

[29] Newberry RC (1995) Environmental enrichment: increasing the biological relevance of captive environments. Applied Animal Behaviour Science 44: 229–243.

[30] Rickard NS, Toukhsati SR, Field SE (2005) The effect of music on cognitive performance: Insight from neurobiological and animal studies. Behavioral and Cognitive Neuroscience Reviews 4: 235–261. 16585799 10.1177/1534582305285869

[31] Moretti DV, Babiloni C, Binetti G, Cassetta E, Dal Forno G, et al (2004) Individual analysis of EEG frequency and band power in mild Alzheimer’s disease. Clinical Neurophysiology 115: 299–308. 14744569 10.1016/s1388-2457(03)00345-6

[32] Huang C, Wahlund LO, Dierks T, Julin P, Winblad B, et al (2000) Discrimination of Alzheimer’s disease and mild cognitive impairment by equivalent EEG sources: a cross-sectional and longitudinal study. Clinical Neurophysiology 111: 1961–1967. 11068230 10.1016/s1388-2457(00)00454-5

[33] Fernández T, Harmony T, Rodríguez M, Bernal J, Silva J, et al (1995) EEG activation patterns during the performance of tasks involving different components of mental calculation. Electroencephalography and Clinical Neurophysiology 94: 175–182. 7536152 10.1016/0013-4694(94)00262-j

[34] Delorme A, Makeig S (2004) EEGLAB: an open source toolbox for analysis of single-trial EEG dynamics including independent component analysis. Journal of Neuroscience Methods 134: 9–21. 15102499 10.1016/j.jneumeth.2003.10.009

[35] Addante RJ, Watrous AJ, Yonelinas AP, Ekstrom AD, Ranganath C (2011) Prestimulus theta activity predicts correct source memory retrieval. Proceedings of the National Academy of Sciences 108: 10702–10707. 10.1073/pnas.1014528108 PMC3127901 21670287

[36] John E, Ahn H, Prichep L, Trepetin M, Brown D, et al (1980) Developmental equations for the electroencephalogram. Science 210: 1255–1258. 7434026 10.1126/science.7434026

[37] Cohen J (1992) A power primer. Psychological Bulletin 112: 155–159. 19565683 10.1037//0033-2909.112.1.155

[38] Utts JM, Heckard RF (2006) Statistical ideas and methods. Belmont, CA: Thomson Brooks/Cole. 361.

[39] Baijal S, Srinivasan N (2010) Theta activity and meditative states: spectral changes during concentrative meditation. Cognitive Processing 11: 31–38. 19626355 10.1007/s10339-009-0272-0

[40] Pfurtscheller G, Stancak A, Neuper C (1996) Post-movement beta synchronization. A correlate of an idling motor area? Electroencephalography and Clinical Neurophysiology 98: 281–293. 8641150 10.1016/0013-4694(95)00258-8

[41] Servít Z, Machek J, Fischer J (1965) Electrical activity of the frog brain during electrically induced seizures. A comparative study of the spike and wave complex. Electroencephalography and Clinical Neurophysiology 19: 162–171. 4157588 10.1016/0013-4694(65)90226-9

[42] Ono K, Baba H, Mori K, Sato K (1980) EEG activities during kindling in frog. International Journal of Neuroscience 11: 9–15. 7390708 10.3109/00207458009147573

[43] Westhoff G, Roth G (2002) Morphology and projection pattern of medial and dorsal pallial neurons in the frog Discoglossus pictus and the salamander Plethodon jordani. The Journal of Comparative Neurology 445: 97–121. 11891656 10.1002/cne.10136

[44] Neary TJ (1990) The pallium of anuran amphibians. In: Jones E, Peters A, editors. Comparative Structure and Evolution of Cerebral Cortex. New York: Plenum Press. 107–138.

[45] Kostowski W (1967) Pharmacological analysis of bioelectrical activity of the central nervous system of amphibia. I. The effect of some cholinergic and anticholinergic drugs on the frog’s EEG. Brain Research 6: 783–785. 6080230 10.1016/0006-8993(67)90137-0

[46] Hobson J, Goin O, Goin C (1968) Electrographic correlates of behaviour in tree frogs. Nature 220: 386–387. 5684884 10.1038/220386a0

[47] Sauseng P, Griesmayr B, Freunberger R, Klimesch W (2010) Control mechanisms in working memory: a possible function of EEG theta oscillations. Neuroscience & Biobehavioral Reviews 34: 1015–1022. 20006645 10.1016/j.neubiorev.2009.12.006

[48] Lynch KS, Crews D, Ryan MJ, Wilczynski W (2006) Hormonal state influences aspects of female mate choice in the túngara frog (*Physalaemus pustulosus*). Hormones and Behavior 49: 450–457. 16277986 10.1016/j.yhbeh.2005.10.001 PMC2581836

[49] Bisazza A (1998) The origins of cerebral asymmetry: a review of evidence of behavioural and brain lateralization in fishes, reptiles and amphibians. Neuroscience & Biobehavioral Reviews 22: 411–426. 9579329 10.1016/s0149-7634(97)00050-x

[50] Walker S (1980) Lateralization of functions in the vertebrate brain: a review. British Journal of Psychology 71: 329–367. 7407442 10.1111/j.2044-8295.1980.tb01750.x

[51] Davidson RJ (1984) Affect, cognition, and hemispheric specialization. In: Izard CE, Kagen J, Zajonc R, editors. Emotions, Cognition, and Behavior. Cambridge, UK: Cambridge University Presss. 320–365.

[52] Davidson RJ (1984) Hemispheric asymmetry and emotion. In: Scherer K, Ekman P, editors. Approaches to Emotion. Hillsdale, NJ: Erlbaum. 39–57.

[53] Davidson RJ, Ekman P, Saron CD, Senulis JA, Friesen WV (1990) Approach-withdrawal and cerebral asymmetry: Emotional expression and brain physiology: I. Journal of Personality and Social Psychology. 58: 330–341. 2319445

[54] Sutton SK, Davidson RJ (1997) Prefrontal brain asymmetry: A biological substrate of the behavioral approach and inhibition systems. Psychological Science 8: 204–210.

[55] Bak P, Tang C, Wiesenfeld K (1987) Self-organized criticality: An explanation of the 1/f noise. Physical Review Letters 59: 381–384. 10035754 10.1103/PhysRevLett.59.381

[56] Bak P, Tang C, Wiesenfeld K (1988) Self-organized criticality. Physical Review A 38: 364–374. 10.1103/physreva.38.364 9900174

[57] Montgomery SM, Betancur MI, Buzsáki G (2009) Behavior-dependent coordination of multiple theta dipoles in the hippocampus. Journal of Neuroscience 29: 1381–1394. 19193885 10.1523/JNEUROSCI.4339-08.2009 PMC2768079

[58] Lee H, Simpson GV, Logothetis NK, Rainer G (2005) Phase locking of single neuron activity to theta oscillations during working memory in monkey extrastriate visual cortex. Neuron 45: 147–156. 15629709 10.1016/j.neuron.2004.12.025

[59] Akre KL, Ryan MJ (2010) Complexity increases working memory for mating signals. Current Biology 20: 502–505. 20206525 10.1016/j.cub.2010.01.021

[60] Sauseng P, Klimesch W, Gruber WR, Birbaumer N (2008) Cross-frequency phase synchronization: a brain mechanism of memory matching and attention. Neuroimage 40: 308–317. 18178105 10.1016/j.neuroimage.2007.11.032

[61] Philiastides MG, Sajda P (2007) EEG-informed fMRI reveals spatiotemporal characteristics of perceptual decision making. Journal of Neuroscience 27: 13082–13091. 18045902 10.1523/JNEUROSCI.3540-07.2007 PMC6673396

[62] Engel AK, Fries P (2010) Beta-band oscillations–signalling the status quo? Current Opinion in Neurobiology 20: 156–165. 20359884 10.1016/j.conb.2010.02.015

[63] Ray WJ, Cole HW (1985) EEG alpha activity reflects attentional demands, and beta activity reflects emotional and cognitive processes. Science 228: 750–752. 3992243 10.1126/science.3992243

[64] Brismar T (2007) The human EEG-physiological and clinical studies. Physiology & Behavior 92: 141–147. 17585964 10.1016/j.physbeh.2007.05.047

[65] Knyazev GG (2007) Motivation, emotion, and their inhibitory control mirrored in brain oscillations. Neuroscience & Biobehavioral Reviews 31: 377–395. 17145079 10.1016/j.neubiorev.2006.10.004

[66] Buzsaki G, Draguhn A (2004) Neuronal oscillations in cortical networks. Science 304: 1926–1929. 15218136 10.1126/science.1099745

